# BRI1 EMS SUPPRESSOR1 genes regulate abiotic stress and anther development in wheat (*Triticum aestivum* L.)

**DOI:** 10.3389/fpls.2023.1219856

**Published:** 2023-08-09

**Authors:** Dezhou Wang, Jinghong Zuo, Shan Liu, Weiwei Wang, Qing Lu, Xiaocong Hao, Zhaofeng Fang, Ting Liang, Yue Sun, Chunman Guo, Changping Zhao, Yimiao Tang

**Affiliations:** ^1^Institute of Hybrid Wheat, Beijing Academy of Agriculture and Forestry Sciences, Beijing, China; ^2^The Municipal Key Laboratory of the Molecular Genetics of Hybrid Wheat, Hubei Collaborative Innovation Center for Grain Industry, Beijing, China; ^3^Agriculture College, Yangtze University, Jingzhou, China

**Keywords:** BRI1 EMS SUPPRESSOR (BES1), expression pattern, abiotic stresses, anther development, thermosensitive genic male sterile

## Abstract

BRI1 EMS SUPPRESSOR1 (BES1) family members are crucial downstream regulators that positively mediate brassinosteroid signaling, playing vital roles in the regulation of plant stress responses and anther development in Arabidopsis. Importantly, the expression profiles of wheat (*Triticum aestivum* L.) *BES1* genes have not been analyzed comprehensively and systematically in response to abiotic stress or during anther development. In this study, we identified 23 *BES1-like* genes in common wheat, which were unevenly distributed on 17 out of 21 wheat chromosomes. Phylogenetic analysis clustered the *BES1* genes into four major clades; moreover, *TaBES1-3A2*, *TaBES1-3B2* and *TaBES1-3D2* belonged to the same clade as Arabidopsis *BES1/BZR1 HOMOLOG3* (*BEH3*) and *BEH4*, which participate in anther development. The expression levels of 23 wheat BES1 genes were assessed using real-time quantitative PCR under various abiotic stress conditions (drought, salt, heat, and cold), and we found that most *TaBES1-like* genes were downregulated under abiotic stress, particularly during drought stress. We therefore used drought-tolerant and drought-sensitive wheat cultivars to explore *TaBES1* expression patterns under drought stress. *TaBES1-3B2* and *TaBES1-3D2* expression was high in drought-tolerant cultivars but substantially repressed in drought-sensitive cultivars, while *TaBES1-6D* presented an opposite pattern. Among genes preferentially expressed in anthers, *TaBES1-3B2* and *TaBES1-3D2* expression was substantially downregulated in thermosensitive genic male-sterile wheat lines compared to common wheat cultivar under sterile conditions, while we detected no obvious differences under fertile conditions. This result suggests that TaBES1-3B2 and TaBES1-3D2 might not only play roles in regulating drought tolerance, but also participate in low temperature-induced male sterility.

## 1 Introduction

The steroid phytohormone brassinosteroid (BR) plays an essential role in a broad range of plant growth and vital physiological processes (Li and Deng, 2005; Wang et al., 2012; Anwar et al., 2018; Feng et al., 2021; Li et al., 2021). BR-INSENSITIVE1 (BRI1)-EMS SUPPRESSOR1 (BES1) and BRASSINAZOLE RESISTANT1 (BZR1) are core transcription factors directly mediating BR signaling (Wang et al., 2012; Chen et al., 2019b; Cui et al., 2019; Cao et al., 2020; Kono and Yin, 2020; Su et al., 2021). A highly conserved DNA binding motif in the N-terminal of BES1-type proteins binds to E-boxes or to BR-response elements to regulate the transcription of target genes (Yin et al., 2005; Wang et al., 2012; Anwar et al., 2018; Su et al., 2021). Recent studies have revealed that BES1/BZR1 family members not only participate in BR signal transduction but also integrate multiple phytohormone signals to control tapetum development and regulate various stress responses (Yin et al., 2002; Wang et al., 2012; Jiang et al., 2015; Saha et al., 2015; Chen et al., 2019a; Chen et al., 2019b; Cui et al., 2019; Planas-Riverola et al., 2019; Setsungnern et al., 2020; Yan et al., 2020; Mecchia et al., 2021; Su et al., 2021; Korwin Krukowski et al., 2022; Sun et al., 2022; Yang et al., 2022).

BES1 and BZR1 possess a conserved BES1-type domain in their N termini and belong to the plant-specific BES1/BZR1 transcription factor family, which consists of eight members in Arabidopsis (*Arabidopsis thaliana* L.), 11 in maize (*Zea mays* L.), 16 in soybean (*Glycine max* L.), 22 in apple (*Malus domestica* L.), and nine in tomato (*Solanum lycopersicum* L.) (Wang et al., 2012; Lachowiec et al., 2018; Li et al., 2018; Li et al., 2019b; Li Y. et al., 2018; Cao et al., 2020; Feng et al., 2021; Su et al., 2021; Sun et al., 2022). The functions of the eight BES1/BZR1 family members in the model species Arabidopsis (BES1, BZR1, BEH1, BEH2, BEH3, BEH4, BMY2, and BMY4) have been well studied (Yin et al., 2005; Guo et al., 2009; Reinhold et al., 2011; Anwar et al., 2018; Lachowiec et al., 2018; Chen et al., 2019b; Otani et al., 2020). BES1 and BZR1 share high sequence similarity and perform essential functions in stress responses. The four BES1 homologs named BEH1, BEH2, BEH3, and BEH4 are partially redundant with BES1 and BZR1 (Chen et al., 2019a; Chen et al., 2019b). Although the single mutants *bes1* and *bzr1* do not exhibit typical BR mutant phenotypes, the quintuple mutant (*bes1 bzr1 beh1 beh3 beh4/qui-1*) is defective in tapetum and microsporocyte development (Chen et al., 2019a; Chen et al., 2019b). The other two members of the BES1/BZR1 family, the β-amylase proteins BMY2 and BMY4, are also associated with BR signaling, controlling shoot growth and development (Thalmann et al., 2019). Anthers of Arabidopsis mutants lacking function of BZR1, BES1, and their homologs are mostly loculeless, indicating that BES1 family members play indispensable roles during anther development (Chen et al., 2019a; Chen et al., 2019b).

Studies on the functions of BES1/BZR1 gene family members have recently been extended to other species. The protein encoded by the drought-responsive BES1 transcription factor gene *TaBZR2* in wheat (*Triticum aestivum* L.) binds to the promoter region of *TaGST1* (*glutathione S-transferase 1*) to activate its transcription, leading to the induction of drought responses (Cui et al., 2019). The liverwort (*Marchantia polymorpha* L.) BES1 member MpBES1 controls cell proliferation and differentiation (Mecchia et al., 2021). Studies on the nine tomato BES1 members revealed that SlBES1.5 (also named SlBZR1) plays a critical role in the BR-mediated regulation of tapetal cell degeneration and pollen development; overexpression of *SlBZR1* increases pollen viability, while the pollen viability of *SlBZR1* loss-of-function mutants decreased compared to that of the wild type (Yan et al., 2020). SlBZR1 also affects tomato anther development by directly binding to the promoter region of the gene *SlRBOH1* (*RESPIRATORY BURST OXIDASE HOMOLOG1*), encoding an NADPH oxidase that produces reactive oxygen species (ROS), leading to tapetum degradation, a decline in pollen viability, decreased pollen germination, and reduced seed number (Yan et al., 2020). These results demonstrate that BES1 members have a profound influence on anther development.

Wheat is a major cereal crop worldwide. Globalization has turned crop productivity into a serious issue affecting food security; hence, improving wheat yield and quality under extreme weather conditions is of vital importance (Khan et al., 2020). Previous studies have shown that BES1/BZR1 family members participate in plant responses to environmental stress (Cui et al., 2019; Kono and Yin, 2020; Liao et al., 2020; Sun et al., 2020; Jia et al., 2021; Van Nguyen et al., 2021), with overexpression of the *TaBZR2* positively regulating wheat drought stress (Cui et al., 2019). Hence, studies on the relationship of wheat BES1 family members with stress responses is very important. The proper development of functional pollen is closely related to wheat yield. Thermosensitive genic male sterile (TGMS) lines are defective in anther development under sterile environmental conditions (low temperature at the meiosis stage) but can reproduce under fertile conditions (moderate temperature during anther development), a characteristic that is widely used in hybrid wheat breeding to aid in wheat production (Bai et al., 2017; Xu et al., 2020). Previous research in Arabidopsis and tomato provides a rationale for the exploration of the role played by TaBES1 in TGMS lines during anther development in wheat (Chen et al., 2019a; Chen et al., 2019b). As a first step, the global identification of all wheat BES1 family members and the investigation of their biological functions will be helpful for identifying stress-related *BES1-like* genes in wheat to improve adaptation to stress (Ahad et al., 2021; Kesawat et al., 2021; Liu et al., 2021). Twenty *TaBZR* genes were recently identified, and the expression profiles of some were explored (Kesawat et al., 2021; Liu et al., 2021). However, the potential functions of TaBES1 members in male sterility remain unknown.

In this study, we performed a global survey of *BES1* genes in common wheat and identified 23 *TaBES1*-*like* genes through comprehensive bioinformatics analysis. We also analyzed their expression patterns under drought, salt, cold, and heat stress conditions. In particular, we tested the expression levels of *TaBES1* members in male sterile lines and common wheat cultivar under sterile and fertile conditions. In subsequent research, we are constructing knockout and overexpression transgene materials specifically targeting *TaBES1-3B2* and *TaBES1-3D2* genes, aiming to elucidate the precise functions of each BES1 gene. Moreover, these materials show significant potential for utilization in wheat breeding. Our study provides new insights into the functions of wheat BES1 members, which will be of vital importance for wheat molecular breeding and wheat production.

## 2 Materials and methods

### 2.1 Identification of BES1 genes

The wheat (*Triticum aestivum*) genome (wheat genome version IWGSCV1.1) and protein sequences were obtained from the Ensembl Plants website (http://plants.ensembl.org/index.html accessed on 11 March 2022). HMMER 3.0 (hidden Markov model, HMM) (http://hmmer.org/) software, BLASTP (https://blast.ncbi.nlm.nih.gov/) method and Pfam (http://pfam.xfam.org/) database searches were used to identify *BES1* genes in *Triticum aestivum*, *Aegilops tauschii*, *Triticum dicoccoides*, *Triticum turgidum*, *Triticum urartu*, and *Hordeum vulgare* using reported Arabidopsis, rice, and maize *BES1* genes as queries. BES1 proteins were verified using HMMER software (E value < 0.01) and the NCBI conserved domain database (https://www.ncbi.nlm.nih.gov/Structure/cdd/wrpsb.cgi). The HMM profile of the BES1 domain was downloaded from the Pfam (PF05687) and WheatOmics (http://wheatomics.sdau.edu.cn/tools/proteinfamily.html) databases. The physical and chemical properties of TaBES1 proteins from the longest transcripts were determined using the ExPASy website (http://web.ExPASy.org/translate/).

### 2.2 Phylogenetic and chromosomal location analysis

MEGA 10.0 software was used to reconstruct a phylogenetic tree of the TaBES1 family using the maximum-likelihood method with the full-length protein sequences. The BES1-like proteins from eight species were divided into subfamilies according to their clustering patterns. Each *TaBES1* gene was mapped onto one of the 21 wheat chromosomes according to the IWGSC RefSeq v1.1 (‘Chinese Spring’) reference genome of the Triticeae Multi-omics Center (http://wheatomics.sdau.edu.cn/). The Ensembl database (http://plants.ensembl.org/biomart/martview/) was used to extract information about chromosome length, and a physical map was drawn using MG2C software v2.1 (http://mg2c.iask.in/mg2c-v2.1/).

### 2.3 Analysis of gene structures, motifs, and promoter cis-regulatory elements

Gene structures were visualized using the Gene Structure Display Server (http://gsds.gao-lab.org/). Conserved motifs of TaBES1 proteins were analyzed using MEME (http://meme-suite.org/index.html). TBtools software (https://github.com/CJ-Chen/TBtools/releases) was then used to analyze and visualize the *TaBES1* gene structures and conserved motifs of TaBES1 proteins. A 2-kb upstream promoter region for each *TaBES1* gene was retrieved from the Ensembl Plants website and then analyzed using PlantCARE (http://bioinformatics.psb.ugent.be/webtools/plantcare/html/), finally the prediction of *cis*-regulatory elements was visualized using TBtools software. Alignment of BES1-domain sequences was performed using MEGA 10 software, and the sequence logo of this domain was created using the weblogo website (http://weblogo.berkeley.edu/).

### 2.4 Predicted microRNA targets and analysis of predicted gene expression

Sequences of *TaBES1* genes were uploaded to the psRNATarget website (https://www.zhaolab.org/psRNATarget/) to search for potential miRNA-mediated regulation based on known miRNAs and gene sequence information. The psRobot website (http://omicslab.genetics.ac.cn/) was used for the analysis of small RNA target predictions, and putative regulatory networks were visualized using Cytoscape software v3.8.2 (https://cytoscape.org/). Expression data based on transcriptome deep sequencing (RNA-seq) and microarray databases of T. aestivum ‘Chinese Spring’ from the WheatOmics database (http://wheatomics.sdau.edu.cn/). To analyze tissue-specific expression patterns, the coding sequence of each gene was submitted to the WheatOmics database to obtain expression data for roots, stems, leaves, spikes, anthers, and grain tissues. Transcripts per million (TPM) values for *TaBES1* genes under drought, salt, cold, and heat stress conditions were also retrieved from the Triticeae Multi-omics Center database and visualized as heatmaps using TBtools software.

### 2.5 Plant materials and stress treatments

To investigate the tissue-specific expression profiles of *TaBES1* genes, the following tissues were collected: root and shoot tissues at the seedling stage, leaves at the tilling stage, spikes and anthers at the full heading stage, and grain at the milking stage, from the wheat cultivar ‘Xiaobaimai’ (XBM), which serves as the original backbone parent wheat variety in northern China. As XBM is known for its sensitivity to abiotic stress at seedling stage (Li L. et al., 2018; Cao et al., 2021), its seeds were placed in Petri dishes containing two pieces of filter paper soaked with water at 23°C under a 16 h light/8 h dark photoperiod; germinated seeds were then transferred to plastic mesh grids at 28°C under a 16 h light/8-h dark photoperiod. Fourteen-day-old XBM seedlings were exposed to drought (25%, [w/v] polyethylene glycol 6000 [PEG-6000]), salt (250 mM NaCl), cold (4°C), or heat (40°C) for 0, 1, 2, 5, 10, or 24 h (Liu et al., 2015; Xu et al., 2020), and they were used for the gene expression analysis.

Seedlings from the cultivars ‘Taiyuan806’ (TY806), ‘Zhongyou9507’ (ZY9507), ‘Yumai8’ (YM8), ‘Xinong318’ (XN318), and ‘Zhongyin6’ (ZY6) were used to test drought resistance. TY806, XN318, and ZY6 were reported to be drought-tolerant, while XBM, ZY9507, and YM8 were considered drought-sensitive cultivars (Li L. et al., 2018; Cao et al., 2021). They were planted in pots containing a mixture of vermiculite and humus soil in equal volumes, and grown under appropriate watering conditions at 70% relative humidity and a temperature of 25°C, with a photoperiod of 16 hours of light and 8 hours of darkness in a climate incubator. When the seedlings reached the 2-leaf stage, watering was withheld for 2 weeks to induce drought stress. Additionally, PEG-induced drought stress was applied to these six wheat varieties. The seeds of the six wheat cultivars were germinated in a dark environment at a temperature of 23°C. After a two-day germination period, uniform seedlings were selected and transplanted into plastic containers filled with a hydroponic solution based on the Hoagland culture method. The growth conditions were maintained at a temperature of 28°C, with a photoperiod of 16 hours of light and 8 hours of darkness. Subsequently, ten-day-old seedlings with two leaves were subjected to PEG-induced drought stress (PEG-6000, 25% w/v) for 24 hours. To assess the impact of drought stress, the superoxide dismutase (SOD) activity, malondialdehyde (MDA) content, and peroxidase (POD) activity of the entire seedlings were measured. These measurements were conducted using the Total Superoxide Dismutase assay kit, Plant Malondialdehyde assay kit, and Peroxidase assay kit, respectively (Jiancheng Bioengineering Institute located in Nanjing, China). All assays were performed according to the manufacturer’s instructions. The expression levels of the other five wheat cultivars were assessed using 14-day-old seedlings under 0, 1, 2, 5, 10, and 24 hours of PEG treatment, which was the same as the treatment given to XBM. Finally, the seedlings of all six wheat cultivars under PEG treatment were used for gene expression analysis. Three independent experiments were performed for each line (Li L. et al., 2018; Cao et al., 2021)

Temperature was found to play a crucial role in controlling the fertility of TGMS lines, with moderate temperature promoting fertility and low temperature inducing sterility during the anther development (Liu et al., 2015; Xu et al., 2020). In this study, TGMS lines BS1453 and BS366 were grown in Beijing (BJ) (moderate temperature fertile environment) and Nanyang (NY) of Henan province (low temperature sterile environment). The common wheat cultivar ‘Jing411’ (J411) was used as a control and exhibited a fertile phenotype in both the BJ and NY environments. Anthers from BJ and NY environments were collected and frozen in liquid nitrogen immediately at each male reproductive development stage of central callose (S6), meiotic (S7), tetrad (S8), and young microspore (S9) (Browne et al., 2018; Xu et al., 2020). After confirming the anther development stages by microscope, anthers at the four developmental stages from BS1453, BS366 and J411 were performed for the following gene expression analysis by qPCR. The pollen starch was subjected to staining using a 10% (v/v) Lugol solution (Lee et al., 2016; Xu et al., 2020). The RNAs of 12 samples (anthers at S9 stage of BS1453, BS366 and J411 under fertile and sterile conditions, two biological replicates per stage of three cultivars) were subjected to 150 bp paried-end sequencing using Novaseq 6000 platform (Illumina), sequencing were performed according to the manufacturer’s standard protocol. Illumina RNA seq was constructed using the method of Külahoglu et al. (Browne et al., 2018; Xu et al., 2020). Each gene’s transcript profile was evaluated using fragments per kilobase of exon per million fragments mapped (FPKM).

### 2.6 RNA extraction and quantitative real-time PCR analysis

Seedlings under various abiotic stress conditions and anthers from sterile and fertile conditions were collected and stored at -80°C. Total RNA was isolated from the samples using TRIzol reagent (Invitrogen) (Pandey et al., 2022). For qPCR, first-strand cDNA was synthesized using a Primer Script RT reagent Kit with gDNA Eraser (TaKaRa). The transcript levels were determined with ChamQ SYBR qPCR Master Mix (Vazyme, Nanjing, China), and the qPCR were conducted in a CFXTM qPCR detection system (Bio-Rad, California, USA). Reaction systems were prepared in 20 μL volumes as follows: 10μL of 2μL Taq Pro Universal SYBR qPCR Master Mix, 6 μL of RNase-free water, 2 μL of cDNA, 1 μL of forward primer, and 1 μL of reverse primer. The primers were designed using Primer Premier 5.0. (**Table S2**). The qPCR reaction conditions were 94°C for 30 s, followed by 40 cycles of 95°C for 5s, 60°C for 30 s. Then a melting curve of 95°C for 15 s, 60°C for 60 s, and 95°C for 15 s was used. *TaACTIN* gene (Gene ID: LOC542814) was performed as the reference gene and the relative gene expression levels were calculated using the 2^-ΔΔCt^ method. Three biological replicates were performed for each treatment, with three technical replicates per sample.

## 3 Results

### 3.1 Identification and chromosomal distribution of BES1 genes in common wheat and its progenitors

Utilizing bioinformatics tools such as HMMER analysis and searches with the BLASTP tool and the Pfam database, we identified 59 putative *BES1-like* genes from the genomes of common wheat and its relatives. Of these genes, 23 were in *Triticum aestivum*, four in red wild einkorn wheat (*Triticum urartu* L.), 13 in wild emmer wheat (*Triticum dicoccoides* L.), 6 in Tausch’s goatgrass (*Aegilops tauschii* L.), and thirteen in durum wheat (*Triticum turgidum* L.), (**Table S1**). According to the gene nomenclature, we named the 23 wheat *BES1-like* genes based on their chromosomal locations and homoeologous genes was named in the same number with different alphabet letters corresponding to their respective subgenomes (**Table S1**). *TaBES1* genes were unevenly distributed across 17 of 21 wheat chromosomes (**Figure S1**). Each chromosome harbored one *TaBES1* gene except chromosomes 3A, 3B, 3D, 6A, and 6B, which each carried two genes. No *TaBES1* gene mapped to chromosomes 1B, 1D, 5A, or 5D. The 23 *TaBES1* genes were almost evenly distributed across the A (8), B (8), and D (6) subgenomes. As listed in **Table S1**, the average amino acid length of the putative TaBES1 proteins was 365 amino acids (aa), ranging from 178 to 686 aa, and TaBES1-4D was the longest protein (686 aa). The predicted molecular weights of TaBES1 proteins ranged from 19.27 kDa (TaBES1-3D1) to 75.47 kDa (TaBES1-4D); all TaBES1 proteins were hydrophobic.

### 3.2 Phylogenetic analysis in common wheat and its progenitors

**Figure 1:**
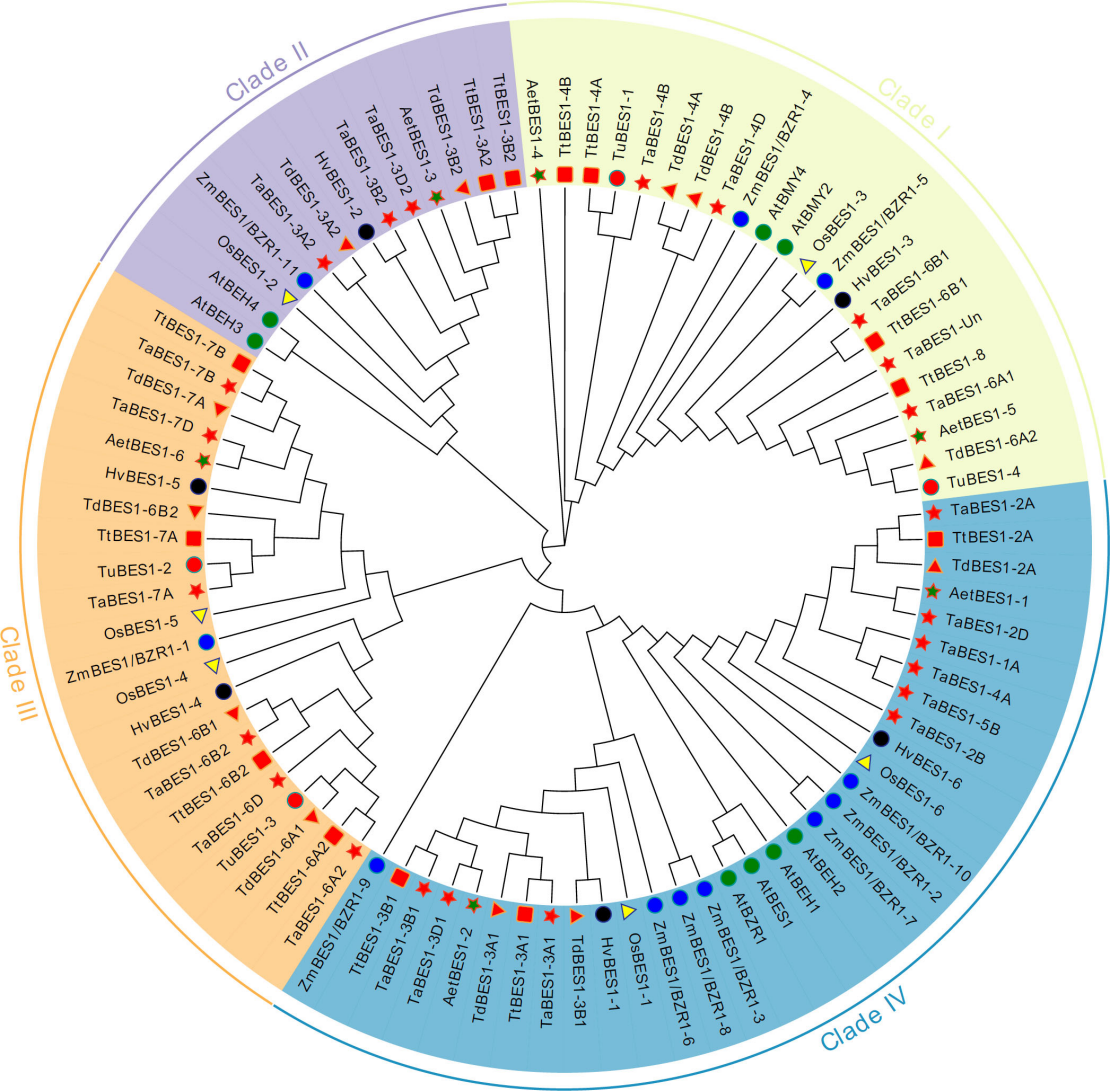
Phylogenetic analysis of BES1 proteins. The maximum-likelihood tree was constructed based on the BES1 proteins from Arabidopsis (At), rice (Os), maize (Zm), wheat (Ta) and its relatives (*Triticum Urartu*, Tu; *Triticum dicoccoides*, Td; *Triticum turgidum*, Tt; and *Aegilops tauschii*, Aet) via Mega 10 software.

We constructed an unrooted phylogenetic tree using the full-length sequences for the 59 BES1 proteins from common wheat and its relatives identified in this study and those of reported BES1 proteins from Arabidopsis (eight), rice (six), and maize (eleven) (**Table S3**). The tree formed four major clades (I-IV) based on the phylogenetic relationship between each BES1-like protein and Arabidopsis BES1 members (**Figure 1**). TaBES1-4B, TaBES1-4D, TaBES1-6A1, TaBES1-6B1, and TaBES1-Un were closely related to AtBMY2 and AtBMY4, which clustered in clade I. TaBES1-3A2, TaBES1-3B2, and TaBES1-3D2 formed a tight clade with Arabidopsis BEH3 and BEH4 within clade II. Clade III contained TaBES1-6A2, TaBES1-6B2, TaBES1-6D, TaBES1-7A, TaBES1-7B, and TaBES1-7D, with no Arabidopsis member. Finally, TaBES1-1A, TaBES1-2A, TaBES1-2B, TaBES1-2D, TaBES1-3A1, TaBES1-3B1, TaBES1-3D1, TaBES1-4A, and TaBES1-5B were closely related to Arabidopsis tapetum development associated proteins BEH1, BEH2, BES1, and BZR1, which all belonged to the largest clade IV.

### 3.3 Gene structure, cis-acting elements, and conserved motifs

We analyzed the exon-intron structure of all wheat *BES1-like* genes by aligning their predicted coding sequences to the wheat genome. *TaBES1* genes from the same subclade shared a common gene structure (**Figure S2A**). Most wheat *BES1* genes contained one intron and two exons; *TaBES1-4A* had two introns and three exons, while *TaBES1-4B*, *TaBES1-4D*, *TaBES1-6A1*, *TaBES1-6B1*, and *TaBES1-Un* belonging to clade I had more than seven introns. The intron and exon number of clade I was far greater than that of the other clades.

We also examined *cis*-regulatory elements within 2-kb fragments of promoter sequence upstream of each *TaBES1* gene. As shown in **Figure S2B**, we identified *cis*-acting elements related to phytohormone signaling, including methyl jasmonate, abscisic acid, salicylic acid, gibberellin, and auxin. We also detected several abiotic stress responsive *cis*-acting elements, such as those associated with wounding, drought, cold, and light.

Proteins belonging to the same subfamily and sharing similar motif compositions are likely to share similar functions, prompting us to characterize functional domains in each TaBES1 protein (Li et al., 2019b). Motif analysis highlighted the high level of conservation of TaBES1 proteins, with motif 1 being shared by all TaBES1 proteins except TaBES1-5B (**Figure S2C**). Moreover, we detected motif 2 in all proteins except TaBES1-6A1, while motif 5 was present in all proteins except TaBES1-3A1, TaBES1-3B1, and TaBES1-3D1. Closely related family members in the phylogenetic tree shared a common motif composition (Li et al., 2019b); we identified motif 10 in members from subclades I, II, and III, while motifs 3, 8, and 9 only existed within subclade I members (**Figure S2C**).

The BES1-type domain is conserved across plant species. An analysis of conserved amino acid residues among wheat BES1-type domains produced results similar to comparable analyses in Arabidopsis, maize, tomato, and other species, illustrating the high conservation of the N terminus, while the C terminus of TaBES1 members was less conserved (**Figure S3A**). BES1 proteins contain a putative nuclear localization sequence, a highly conserved N-terminal region, a BIN2 (BR-INSENSITIVE 2) phosphorylation domain, a PEST motif, and a C-terminal structure (Cao et al., 2020). Similar to other species, sequence logo analysis showed that the N-terminal BES1-type domain was highly conserved between Arabidopsis and wheat BES1 family members (**Figure S3B**).

### 3.4 Regulatory network between putative miRNAs and their target TaBES1 genes

**Figure 2:**
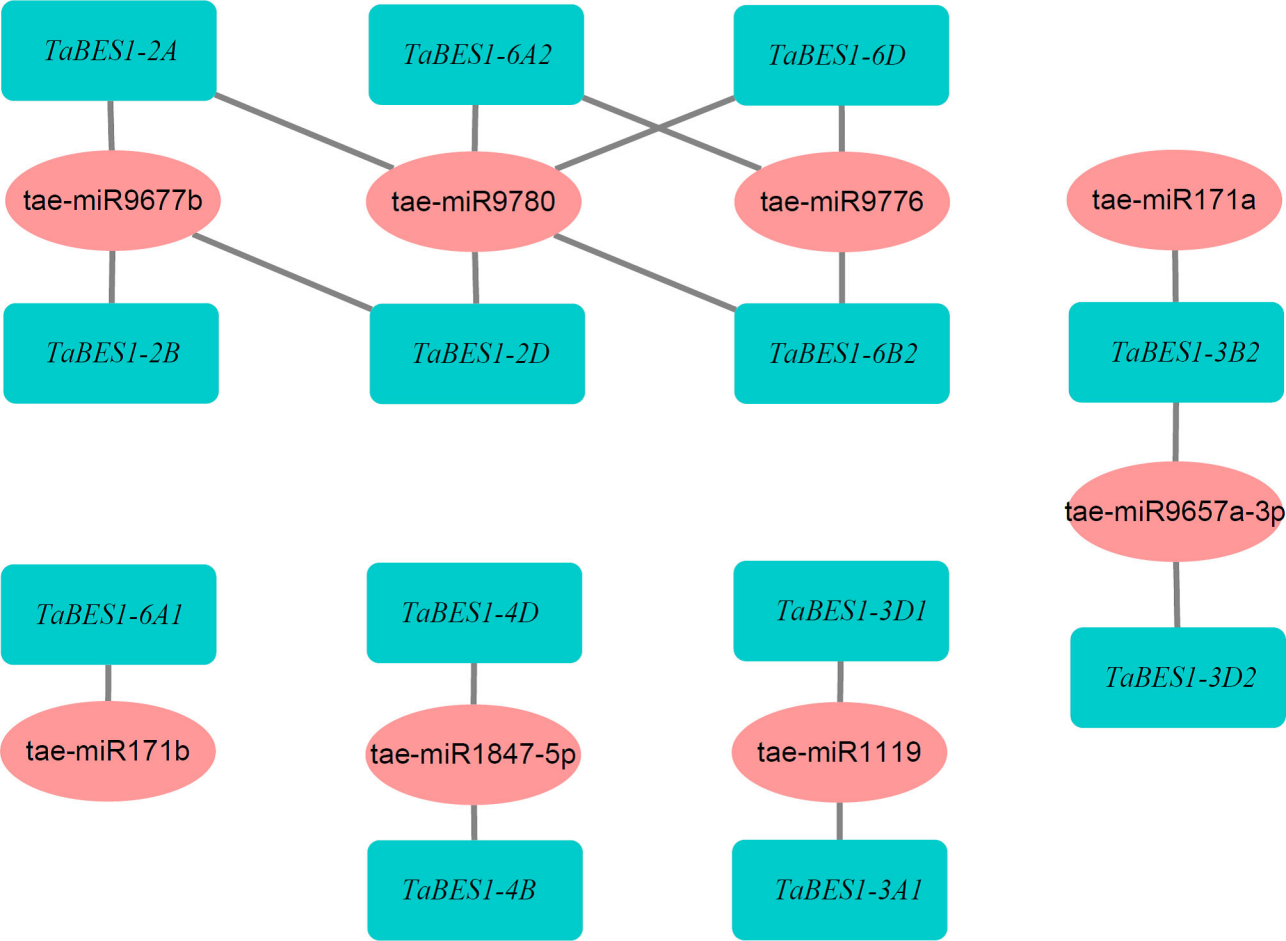
A putative network of wheat miRNAs and targeted *TaBES1* genes.

To investigate the potential regulation of *TaBES1* genes by small regulatory RNAs, we uploaded all 23 *TaBES1* sequences to the wheat miRNA database; which predicted that eight wheat miRNAs have the capacity to regulate *TaBES1* transcript levels, based on their high sequence complementarity (**Figure 2**). Most *TaBES1* genes were regulated by only one miRNA, although *TaBES1-2A*, *TaBES1-2D*, *TaBES1-3B2*, *TaBES1-6A2*, *TaBES1-6B2*, and *TaBES1-6D* were each regulated by two miRNAs. Notably, Tae-miRNA9780 targeted the transcripts of five *TaBES1* genes (*TaBES1-2A*, *TaBES1-2D*, *TaBES1-6A2*, *TaBES1-6B2*, and *TaBES1-6D*), suggesting its importance during BR signal transduction. The miR171, miR1119, miR9657, miR9776, and miR9780 families have been reported to play roles in plant stress responses, with the miR171, miR9657, and miR9677 families being also associated with anther development (Bai et al., 2017; Shi et al., 2018; Sun et al., 2018; Li et al., 2019a; Zeeshan et al., 2021; Zhang et al., 2022).

### 3.5 Tissue-specific expression patterns of TaBES1 genes

We obtained initial tissue expression data from the WheatOmics database. The expression levels of *TaBES1* genes varied across tissues and developmental stages (**Figure S4**). Expression levels of nine of the 23 *TaBES1* family members were extremely low during all developmental stages and in all tissues. By contrast, *TaBES1-3A2*, *TaBES1-3B2*, and *TaBES1-3D2* on chromosome 3 showed higher expression levels during the whole life cycle (**Figure S4**). We confirmed the expression patterns of *TaBES1* genes in roots, stems, leaves, spikes, anthers, and seed from XBM by qPCR, which revealed that expression levels in root tissues are relatively higher than those in other tissues (**Figure S5**). *TaBES1-1A* belongs to the same subgroup as *SlBZR1*, which also contains Arabidopsis *BES1*, *BZR1*, and *BEH1* involved in anther development (**Figure 1**). According to the wheat-expression databases and qPCR results, *TaBES1-3A2, TaBES1-3B2*, *TaBES1-3D2*, *TaBES1-4B*, and *TaBES1-4D* exhibited relatively higher expression levels in anthers than in other tissues and were therefore considered anther-preferential genes (**Figure S5**).

### 3.6 Expression patterns of TaBES1 genes under drought, salt, cold, and heat stress conditions

**Figure 3:**
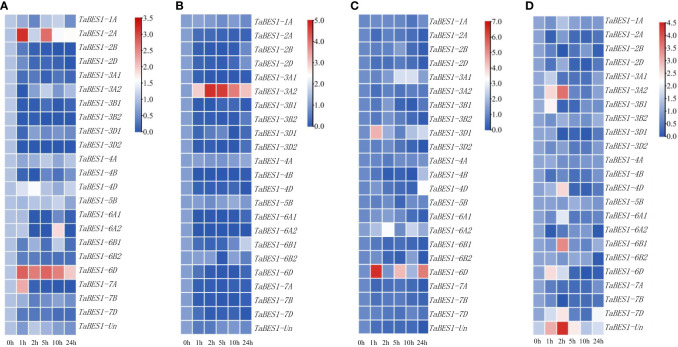
Expression profiles of *TaBES1s* under abiotic stresses: **(A)** drought (PEG6000), **(B)** salt, **(C)** cold and **(D)** heat stress conditions using qPCR. A heatmap of the relative expression of all the *TaBES1* genes. The color key (blue to red) represent the relative gene expression values as fold change. For each gene, the 0 h treatment expression value was set as 1.0. Relative expression of each *TaBES1* gene was normalized to *TaACTIN*.

To determine how *TaBES1* transcription responds to abiotic stresses, we downloaded expression data for *TaBES1* genes in plants exposed to drought, salt, cold, or heat from the WheatOmics database; most *TaBES1* genes were substantially downregulated under these abiotic stress conditions (**Figure S6**). To validate the expression patterns of *TaBES1* genes, we conducted qPCR using total RNA extracted from fourteen-day-old XBM seedlings subjected to drought, salt, cold, and heat abiotic stresses for 0, 1, 2, 5, 10, 24 h. Finally, we observed a high degree of congruence between qPCR and RNA-seq data, with the transcript levels of most genes being affected by stress treatments visualized as heatmaps using TBtools software with log scale base as 2 and log with as 1 (**Figure 3**). Drought stress considerably downregulated the expression levels of most *TaBES1* genes, with the exception of *TaBES1-2A*, *TaBES1-6D*, and *TaBES1-7A*, which were upregulated under the same conditions (**Figure 3A**). In response to salt stress, the expression levels of all family members tended to decline, except *TaBES1-3A2*, which was considerably upregulated (**Figure 3B**). The expression levels of almost all *TaBES1* genes were very low upon exposure to cold stress, while *TaBES1-3D1* and *TaBES1-6D* exhibited an upregulation after low-temperature treatment (**Figure 3C**). The expression of *TaBES1-3A2, TaBES1-6B1*, *TaBES1-6D*, *TaBES1-7D*, and *TaBES1-Un* was induced at the beginning of heat treatment, while that of the other *TaBES1* genes was substantially suppressed (**Figure 3D**).

### 3.7 Drought stress response in drought-tolerant and drought-sensitive wheat cultivars

To further elucidate the drought resistance level of each wheat cultivar at the seedling stage, we conducted both water-withhold treatment and PEG-induced drought stress on the six cultivars. The experimental design included a well-watered control group and a drought stress group where water was withheld, validating the previously reported levels of drought resistance. Specifically, TY806, XN318, and ZY6 were found to exhibit drought tolerance, while XBM, ZY9507, and YM8 were identified as drought-sensitive cultivars (Li L. et al., 2018; Cao et al., 2021) (**Figure S8A**). These findings were further confirmed by the application of PEG treatment, which resulted in significantly higher levels of SOD values in the three drought-resistant cultivars compared to the sensitive cultivars. Similarly, POD values exhibited a similar trend to that of the SOD values. Furthermore, the MDA values in the three drought-resistant cultivars were significantly lower than those observed in the sensitive cultivars (**Figure S8B**). Based on these significant findings, the aforementioned three cultivars (TY806, XN318, ZY6) were classified as drought-resistant, whereas the remaining three cultivars (YM8, ZY9507, XBM) were categorized as drought-sensitive.

**Figure 4:**
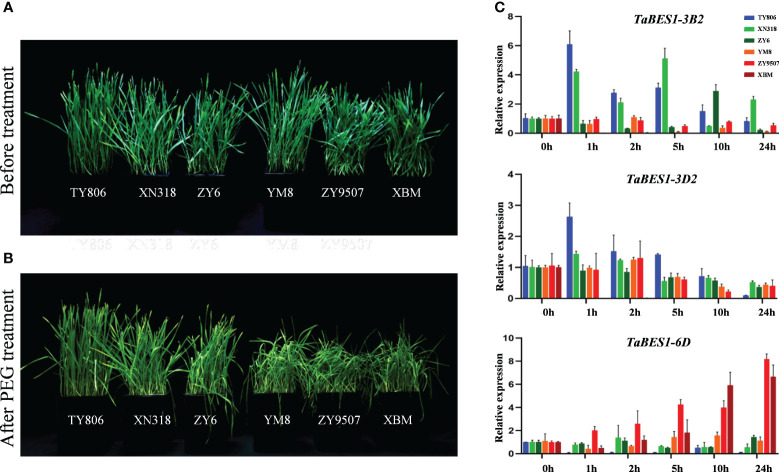
**(A)** Phenotypes of drought tolerant (TY806, XN318, ZY6) and sensitive cultivars (YM8, ZY9507, XBM) at the seedling stage under well-watered control conditions and **(B)** PEG-induced stress conditions (PEG-6000, 25% w/v). **(C)** The expression patterns of *TaBES1-3B2, TaBES1-3D2* and *TaBES1-6D* under 0, 1, 2, 5, 10, 24 h PEG treatment in drought-tolerant (TY806, XN318, and ZY6) and drought-sensitive cultivars (XBM, ZY9507, and YM8). For each gene, the 0 h treatment expression value was set as 1.0. Relative expression of each *TaBES1* gene was normalized to *TaACTIN*.

We further investigated the roles of *TaBES1* members in response to drought stress by measuring their expression profiles in drought-tolerant and drought-sensitive cultivars. Wheat cultivar XBM is stress-sensitive at the seedling stage (Li L. et al., 2018; Cao et al., 2021); we determined that most *TaBES1* genes are considerably suppressed in response to drought stress (**Figure 3A**). By contrast, the expression patterns of *TaBES1-2A*, *TaBES1-3B2*, *TaBES1-3D2*, *TaBES1-6B1*, *TaBES1-6D*, and *TaBES1-7D* were different in the drought-tolerant cultivar TY806 from those in XBM (**Figure S7**). We observed similar expression profiles for these six genes in three drought-sensitive cultivars (XBM, ZY9507, and YM8) and three drought-tolerant cultivars (TY806, XN318, and ZY6) at the seedling stage (Li L. et al., 2018) (**Figures 4A, B**).In general, we detected no obvious commonality in the expression patterns of *TaBES1-2A*, *TaBES1-6B1*, or *TaBES1-7A* between drought-tolerant and -sensitive cultivars (**Figure S8C**). The relative expression levels of *TaBES1-3B2* in drought-tolerant cultivars showed a substantial elevation compared to those measured in drought-sensitive cultivars (**Figure 4C**). *TaBES1-3B2* is the homolog of the positive drought regulator gene *TaBZR2* (*TaBES1-3D2*), which positively regulates the drought stress response(Cui et al., 2019). Thus, we hypothesize that TaBES1-3B2 might have a role in drought signaling, and its function should be investigated in more detail future experiments. The expression patterns of *TaBES1-6D* were different among drought-sensitive and drought-tolerant cultivars; *TaBES1-6D* expression was greatly upregulated in drought-sensitive cultivars but downregulated in drought-tolerant cultivars. We therefore proposed that TaBES1-6D is a negative regulator in the response to drought stress.

### 3.8 Expressions profiles of anther-preferential TaBES1 genes in TGMS wheat anthers

**Figure 5:**
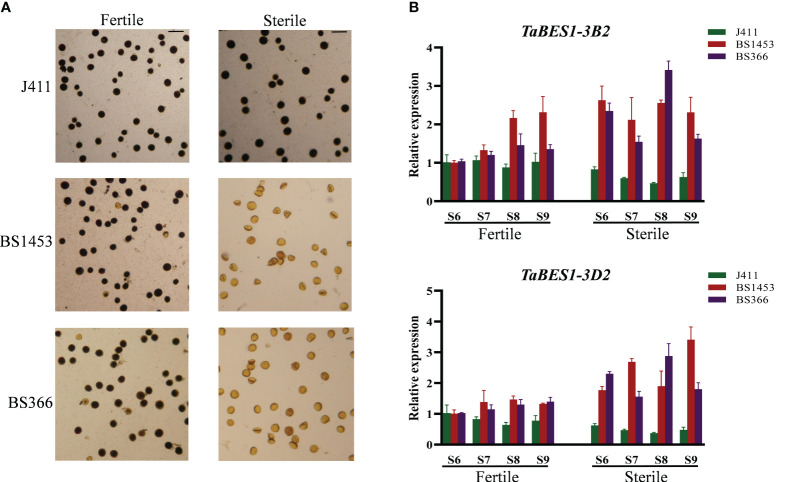
**(A)** Morphology of pollen grains stained with Lugol’s iodine in TGMS lines (BS1453 and BS366) and common wheat (J411) in the moderate temperature fertile conditions and low temperature sterile conditions. Bars=100 μm. **(B)** The expression patterns of *TaBES1-3B2* and *TaBES1-3D2* in TGMS lines (BS1453 and BS366) and common wheat cultivar (J411) in moderate temperature fertile conditions and low temperature sterile conditions. For each gene, the S6 stage under fertile condition expression value was set as 1.0. Relative expression of each *TaBES1* gene was normalized to *TaACTIN*.

Previous studies have reported that BES1 members play roles in anther development (Chen et al., 2019a; Chen et al., 2019b). Therefore, besides the responses to different abiotic stresses, we also explored the potential functions of anther-preferential *TaBES1* genes. Using data from the wheat expression database and qPCR results, we determined the expression levels of anther-preferential wheat *BES1* genes (*TaBES1-1A*, *TaBES1-3A2, TaBES1-3B2*, *TaBES1-3D2*, *TaBES1-4B*, and *TaBES1-4D*) in sterile lines and a common wheat cultivar at the S6~S9 anther developmental stages (Browne et al., 2018), which are extremely important for regulation of anther fertility (Xu et al., 2020). In the case of anthers of TGMS lines under sterile conditions (NY), the sensitive period of anther development aligns with a low-temperature environment, leading to significant inhibition in the development of TGMS anthers and a low seed setting rate. Conversely, under fertile conditions (BJ), anther development is delayed compared to the sterile conditions, occurring in a more favorable moderate-temperature environment, resulting in normal seed setting (Li et al., 2006; Bai et al., 2017; Xu et al., 2020) (**Figure S9A**). Under moderate temperature fertile conditions, the pollen morphology of TGMS lines was similar to the common wheat cultivar, with almost full filled starch; under sterile (low temperature) conditions, however, the pollen of TGMS lines nearly no starch-filled, while the common wheat cultivar pollen was still comparable to that seen under fertile conditions (**Figures 5A**, **S9A**).

In general, no substantial changes were detected in expression levels between the TGMS lines and the common wheat cultivar under fertile conditions (**Figure 5B**); however, we detected substantial differences between the TGMS lines and the common wheat cultivar under sterile (low temperature) conditions (**Figure 5B**), especially for the expression patterns of *TaBES1-3B2* and *TaBES1-3D2* (**Figure 5B**). Indeed, *TaBES1-3B2* expression showed no considerable difference in the common wheat cultivar between sterile and fertile conditions merely with a slight decline under sterile condition, whereas in the TGMS lines, *TaBES1-3B2* expression was higher under sterile conditions as compared to fertile conditions. Notably, the expression pattern of *TaBES1-3D2* was similar to that of *TaBES1-3B2*, showing a considerable rise in expression levels in the TGMS lines under low-temperature conditions. Then the transcriptome analysis was further performed to investigate the expression patterns of *TaBES1s* in TGMS lines and common wheat cultivar (**Figure S10**), and a high degree of congruence was observed between qPCR and RNA-seq results. Generally, in the common cultivar, *TaBES1-3B2* and *TaBES1-3D2* expression showed no considerable difference under fertile and sterile conditions; however, under sterile conditions, the transcript levels of *TaBES1-3B2* and *TaBES1-3D2* were substantially higher than the fertile conditions in TGMS lines.

## 4 Discussion

BRs are necessary throughout the plant life cycle. BES1 family members are widely distributed in plants and play a vital role in BR signaling associated with various abiotic stress responses, cell proliferation and differentiation, seed germination, tapetal cell degeneration, and pollen development (Chen et al., 2019a; Chen et al., 2019b; Yan et al., 2020). Common wheat (*Triticum aestivum*) is an allohexaploid plant, with *Triticum urartu* as the A genome donor, *Aegilops speltoides* as the B genome donor, and *Aegilops tauschii* as the D genome donor. It is thought that *T. urartu* (AA) first hybridized with *A. speltoides* (BB) to form wild emmer wheat (*Triticum dicoccoides*; AABB); *T. dicoccoides* (AABB) then hybridized with *A. tauschii* (DD) to produce common wheat (AABBDD) (Pont et al., 2019). We analyzed the *BES1* gene family in common wheat and its relatives, and found that gene number was largely positively associated with species ploidy level. Wheat appears to contain more *BES1* genes than rice, maize, or other graminaceous crops, and the 23 wheat *BES1* genes are almost evenly distributed across the A, B, and D subgenomes. Among wheat and its relatives, the largest number of *BES1-like* genes were found mapped to group-3 chromosomes, group-6 chromosomes. Interestingly, the number of exons on group-3 chromosomes was generally two, moreover, the synteny analysis showed that the genes on group-3 chromosomes might be relatively conserved over long evolutionary periods among plant species (**Figure S11**).

Studies on BES1 family members have been reported in many species; however, knowledge on the functions of wheat BES1 transcription factors is very limited. Phylogenetic analysis revealed that the 23 wheat BES1 family members can be classified into four subgroups. Relatively conservative TaBES1-3A2, TaBES1-3B2, and TaBES1-3D2 belonged to the same clade as Arabidopsis BEH3 and BEH4, which participate in anther development. Predictions from the wheat-expression database and qPCR results showed that all *TaBES1* genes were highly expressed in roots, with *TaBES1-1A*, *TaBES1-3A2, TaBES1-3B2*, *TaBES1-3D2*, *TaBES1-4B*, and *TaBES1-4D* displaying relatively high expression levels in anthers. Correspondingly, the transcriptome data also showed that *TaBES1-3A2, TaBES1-3B2*, *TaBES1-3D2*, with relatively higher expression levels among all the *TaBES1-like* genes.

BES1 proteins play crucial roles in plant adaptation to environmental stress (Li and Deng, 2005; Anwar et al., 2018; Cui et al., 2019; Liao et al., 2020; Sun et al., 2020); moreover, most *TaBES1* genes displayed root-specific expression. Hence we examined the expression profiles of *TaBES1* genes in response to various stress conditions. The expression of wheat *BES1* genes was induced by drought, salt, cold, and heat treatments, with most *TaBES1* genes exhibiting downregulated expression after stress treatment. Six *TaBES1* genes showed different expression patterns between drought-tolerant and -sensitive cultivars; we confirmed their potential functions in drought stress responses by determining their expression levels in three drought-tolerant lines and three drought-sensitive lines. We identified *TaBES1-3B2*, *TaBES1-3D2*, and *TaBES1-6D* as drought-related genes. The expression levels of *TaBES1-3D2* were high in three drought-tolerant cultivars, especially in the early stages of drought stress, while *TaBES1-3D2* expression declined in drought-sensitive lines during stress. Our study therefore confirmed the function of TaBZR2/TaBES1-3D2 in wheat drought tolerance (Cui et al., 2019). The *TaBES1-3D2* homolog on chromosome 3B is *TaBES1-3B2*, which has a potential function in drought-tolerance; however, another homologous gene, *TaBES1-3A2*, appears to have no obvious relation to drought tolerance.

Previous studies have shown that the miRNA171 family plays roles in responses to biotic and abiotic stresses (Hwang et al., 2011); moreover, tae-miR171a is predicted to enhance salt tolerance based on its substantial downregulation seen in RNA-seq datasets (Zeeshan et al., 2021). tae-miR171a might also mediate the male-sterile signaling pathway, possibly participating in anther development, since tae-miR171a is downregulated in wheat male-sterile lines (Bai et al., 2017). The miRNA1119 family is associated with drought stress, with tae-miR1119 being predicted to play critical roles in regulating wheat drought tolerance (Shi et al., 2018). tae-miR9657 may regulate salt tolerance by enhancing the expression of MYB-related transcription factors (Zeeshan et al., 2021); RNA-seq analysis also indicated that tae-miR9657a-3p is highly abundant in wheat male-sterile lines (Bai et al., 2017). Li et al. and Han et al. both reported that tae-miR9677b levels are lower in male-sterile lines compared to fertile lines (Li et al., 2019a), which might suggest a function in anther development. The gene regulated by miR9776 encoding lipoxygenase plays essential roles in lipid metabolism and confers the osmotic stress response (Zhao et al., 2020), while tae-miR9780 might be involved in the response to abiotic stress (Zhang et al., 2022). Moreover, RNA-seq analysis demonstrated that tae-miR171a displays differential abundance in the wheat TGMS line under both sterile and fertile conditions (Bai et al., 2017); tae-miR171a is believed to be involved in the regulation of wheat male sterility. *TaBES1-3B2* was predicted to be a target gene of tae-miR171a, suggesting a potential role in male sterility. Whereas, the potential functions those miRNAs might have on the regulation of drought stress and anther development still needed further experiments to be proved.

Wheat lines overexpressing *TaBZR2*/*TaBES1-3D2* exhibit improved drought tolerance, while *TaBZR2*/*TaBES1-3D2* RNA interference (RNAi) lines display a drought-sensitive phenotype (Cui et al., 2019). TaBES1-3B2 and TaBES1-3D2 share high sequence identity, suggesting a similar function in the drought stress response. Moreover, our small RNA target prediction analysis revealed that *TaBES1-3B2* is a predicted target gene of tae-miR171a, which confers drought memory to wheat plants (Hwang et al., 2011; Yue et al., 2022); this result suggests that TaBES1-3B2 might be involved in drought stress adaptation. *TaBES1-6D* exhibited the expression pattern opposite to that of *TaBES1-3B2* and *TaBES1-3D2*, suggesting it is a negative regulator of drought response. *TaBES1-6D* was predicted to be the target gene of tae-miR9776 and tae-miR9780, which are both associated with plant osmotic stress signaling pathways (Zeeshan et al., 2021; Zhang et al., 2022), indicating that TaBES1-6D is probably related to stress responses.

Besides their regulation of drought responses, BES1 members participate in anther developmental in several species; for example, tomato SlBZR1 directly binds to the *SlRBOH1* promoter, leading to pollen defects. While TGMS lines also display a pollen defect phenotype, they greatly benefit wheat hybrid breeding and production. Hence, we explored the potential contribution of *TaBES1* genes to TGMS during anther developmental stages. We determined the expression profiles of six genes preferentially expressed in anthers (*TaBES1-1A, TaBES1-3A2, TaBES1-3B2*, *TaBES1-3D2*, *TaBES1-4B* and *TaBES1-4D*) in sterile lines and common wheat cultivar, as well as under fertile and sterile conditions. The expression levels of *TaBES1-3B2* and *TaBES1-3D2* showed large differences in sterile lines compared to the common wheat cultivar. Under fertile conditions, we detected no substantial change between sterile lines and common wheat cultivar. However, under the sterile, low-temperature environment, sterile lines displayed considerably higher expression levels of *TaBES1-3B2* and *TaBES1-3D2*, while those of common wheat cultivar greatly decreased. RNA-seq analysis revealed the expression profiles of *TaBES1-3B2* and *TaBES1-3D2* were basically congruent with qPCR analysis. No substantial changes were observed between sterile lines and common wheat cultivar under fertile conditions, while the expression levels of *TaBES1-3B2* gene was highly increased under sterile conditions in TGMS lines comparing with common wheat cultivar. The phylogenetic analysis illustrated that TaBES1-3B2 and TaBES1-3D2 were closely to Arabidopsis tapetum and microsporocyte development proteins BEH3 and BEH4. In addition, the synteny analysis revealed that *AtBEH3* represented the orthologous gene of *TaBES1-3A2*, *TaBES1-3B2* and *TaBES1-3D2*. Moreover, both the qPCR analysis and transcriptome results indicated *TaBES1-3B2* and *TaBES1-3D2* exhibited high expression levels in the TGMS lines under sterile conditions, which implies a plausible role of these genes in low temperature-induced wheat male sterility. Collectively, our investigation provides a fundamental understanding of wheat male sterility and molecular breeding. Nonetheless, the precise functions and mechanisms of these genes in male sterility require further exploration through the construction of gene knocking-out lines in common wheat.

## 5 Conclusion

Previous studies have demonstrated the functions of BES1 proteins in response to various stresses and their roles in anther development in Arabidopsis. The current study confirmed the potential roles of BES1 members under abiotic stress conditions and during anther development in common wheat. We identified 23 *TaBES1* family members, and their expression profiles indicated that *TaBES1-3B2*, *TaBES1-3D2*, and *TaBES1-6D* might be drought-related genes. Furthermore, *TaBES1-3B2* and *TaBES1-3D2* might play roles in low temperature-induced male sterility signaling pathways. Taken together, these results suggest that TaBES1-3B2 and TaBES1-3D2 are not only involved in drought tolerance, but also possibly participate in the regulation of low temperature-induced male sterility. These results further our understanding of the molecular mechanisms underlying TaBES1 members in regulating plant growth and development.

## Data availability statement

The data presented in the study are deposited in the National Center for Biotechnology Information (NCBI) Sequence Read Archive (SRA) repository, accession number SAMN35358019-SAMN35358030 and the BioProject ID PRJNA976038.

## Author contributions

YT designed the experiment, SL and JZ carried out the experiment, JZ wrote the paper, DW contributed to data analysis. SL, WW, XH, ZF, TL, QL, YS, CG, and CZ participated in field trials. All authors contributed to the article and approved the submitted version.
