# Supplementary material for: BRI1 EMS SUPPRESSOR1 genes regulate abiotic stress and anther development in wheat (Triticum aestivum L.)
Source: Front Plant Sci. 2023 Aug 9;14:1219856. doi: 10.3389/fpls.2023.1219856 (PMC10446898; doi:10.3389/fpls.2023.1219856)
Supplement: Supplementary file 2 [file DataSheet_2.docx]

Figure S1. Chromosomal distribution of the identified *TaBES1* genes across the wheat genome, with chromosome numbers listed above chromosomes, chromosome size listed on the left side of the figure in megabases (Mb).

b

c

a

Figure S2. Phylogenetic relationships and gene structures (a), conserved motifs (b) and cis-acting regulatory elements (c) of BES1 gene family members in common wheat.

a


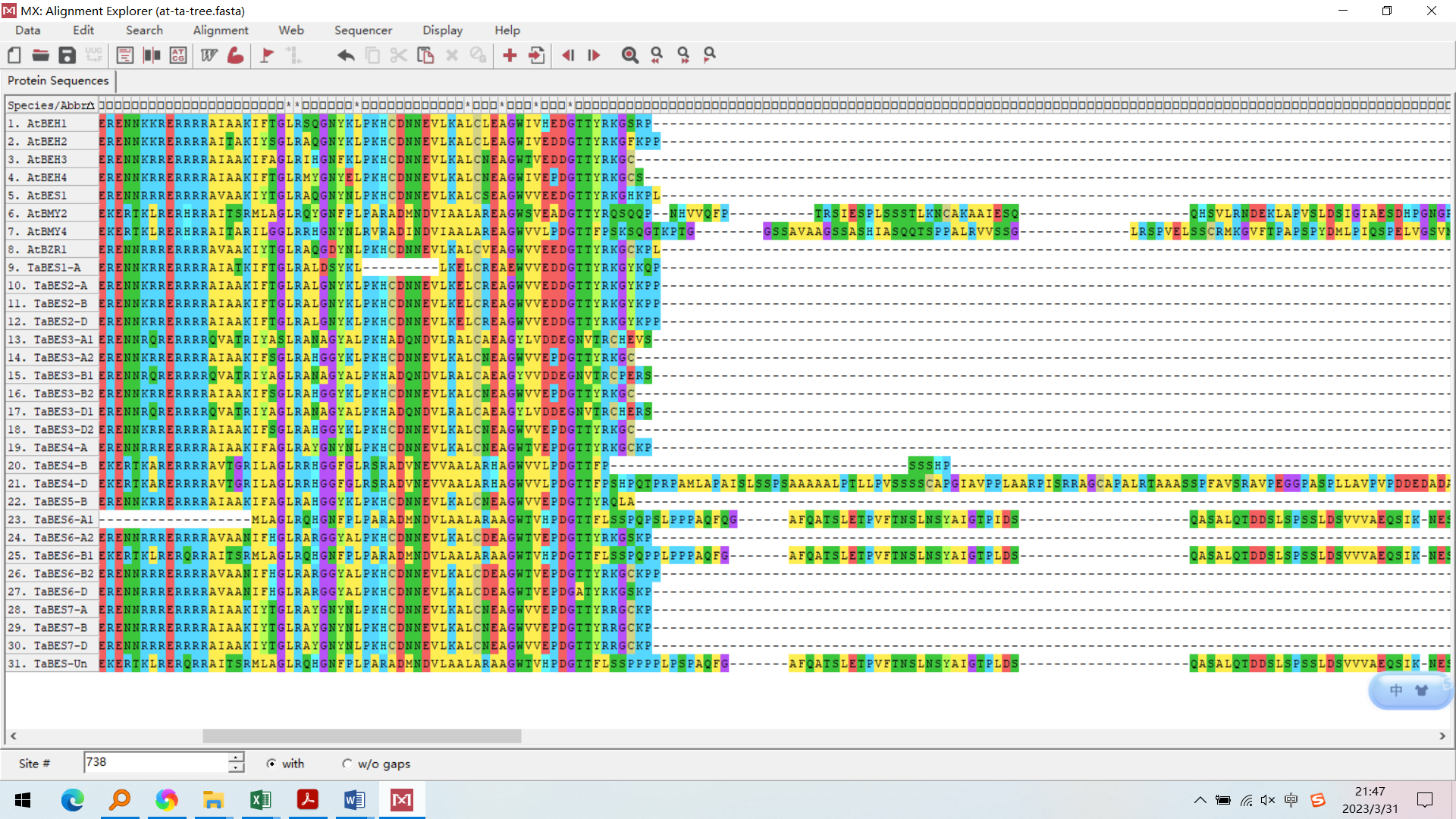


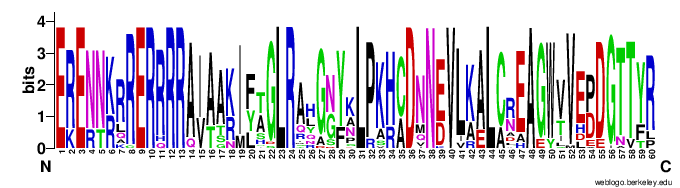


b

Figure S3. (a) Multiple sequence alignment of conserved protein sequence analysis of Arabidopsis and wheat. (b) Sequence logos of BES1-type domain in Arabidopsis and wheat.

Figure S4. Heatmap of the *TaBES1s* expression profiles of the five tissue at different development stages. Heatmap was generated using log_2_(TPM) values.

Figure S5. Heatmap of the *TaBES1s* expression profiles of the five tissue by qPCR. The color key (blue to red) represent the relative gene expression values as fold change. For each gene, the root tissue expression value was set as 1.0. Relative expression of each *TaBES1* gene was normalized to *TaACTIN*.

Figure S6. Heatmap of the *TaBES1s* expression profiles of under various abiotic stresses. Heatmap was generated using log_2_(TPM) values.

Figure S7. Heatmap of the *TaBES1s* expression profiles under drought stress of TY806. The color key (blue to red) represent the relative gene expression values as fold change. For each gene, the 0 h treatment expression value was set as 1.0. Relative expression of each *TaBES1* gene was normalized to *TaACTIN*.

Figure S8. (a) Phenotypes of drought tolerant (TY806, XN318, ZY6) and sensitive cultivars (YM8, ZY9507, XBM) at the seedling stage under well-watered control conditions and drought stress conditions (without water for 2 weeks for drought treatment), and the survival rate after drought stress (b). (c). Expression patterns of *TaBES1-2A*, *TaBES1-6B1* and *TaBES1-7A* under 0, 1, 2, 5, 10, 24 h PEG treatment in drought-tolerant (TY806, XN318, ZY6) and drought-sensitive (XBM, ZY9507, YM8) cultivars. For each gene, the 0 h treatment expression value was set as 1.0. Relative expression of each *TaBES1* gene was normalized to *TaACTIN*.

Figure S9. (a) Morphology of spikes and seed setting rate of TGMS lines (BS1453 and BS366) and common wheat (J411) in the moderate temperature fertile conditions and low temperature sterile conditions. (b) Expression patterns of anther-preferential *BES1* genes except *TaBES1-1A*, *TaBES1-3A2*, *TaBES1-4B*, *TaBES1-4D* between TGMS line BS1453 and common wheat cultivar J411. For each gene, the S6 stage under fertile condition expression value was set as 1.0. Relative expression of each *TaBES1* gene was normalized to *TaACTIN*.

Figure S10. Synteny analysis of BES1 genes among Arabidopsis (At), rice (Os), wheat (Ta), and its relatives (Tu, Aet, Td). The gray lines indicated the collinear blocks within these six species genomes, and the syntenic *BES1* gene pairs were highlighted with the blue lines.


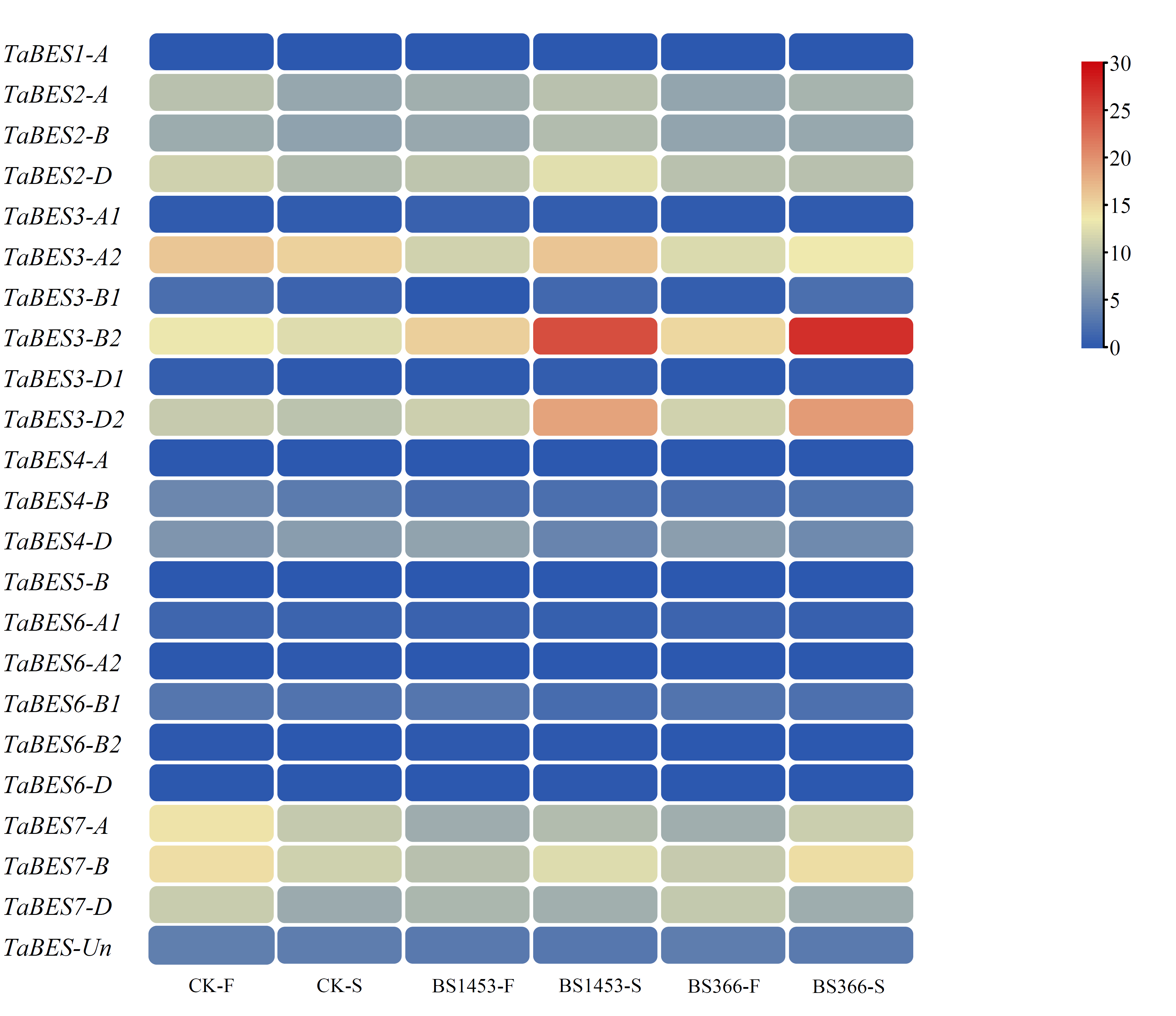


Figure S11. Heatmap of transcript information of *TaBES1s* genes measured in FPKM. The anther of common wheat cultivar J411 at S9 stage was selected as control under fertile (F) and sterile (S) conditions. Anthers of two TGMS lines BS1453 and BS366 under F and S conditions were measured and expression profiles of all the identified *TaBES1s* genes were exhibited with heatmap.
